# Supplementary material for: Cell Type- and Sex-Specific Dysregulation of Thyroid Hormone Receptors in Placentas in Gestational Diabetes Mellitus
Source: Int J Mol Sci. 2020 Jun 5;21(11):4056. doi: 10.3390/ijms21114056 (PMC7313460; doi:10.3390/ijms21114056)
Supplement: Supplementary file 1 [file ijms-21-04056-s001.zip › supplementary material/supplementary material.docx]

**Identification of THR expressing cells in the decidua with double immunofluorescence**

THRα/β + HLA-G double fluorescence staining was used for the discrimination between extravillous trophoblast cells (HLA-G positive) and decidual stromal cells (HLA-negative).

HLA-G expression in the decidua of GDM placentas is shown in green (Figure S1A). THRα1 expression is shown in red (Figure S1B). Triple filter excitation shows that EVTs (HLA-G positive) as well as decidual stromal cells (HLA-G negative) express THRα1 (S1C). In normal control decidual tissue EVTs were also marked with HLA-G in green (Figure 6S1C) and THRα1 is marked in red (Figure S1D). Triple filter excitation (Figure S1E) was used to discriminate between THRα1 expressing cells that co-express HLA-G (EVT) and decidual stromal cells, which do not express HLA-G.

For identification of THRβ1-expressing cells in the decidua, the same approach as for THRα1 was used: HLA-G expression in the decidua of GDM placentas is shown in green (Figure S2A). THRβ1 expression is shown in red (Figure S2B). Triple filter excitation shows that EVTs (HLA-G positive) as well as decidual stromal cells (HLA-G negative) express THRβ1 (S2C). In normal control decidual tissue, EVTs were also marked with HLA-G in green (Figure S2C) and THRβ1 is marked in red (Figure S2D). Triple filter excitation (Figure S2E) was used to discriminate between THRβ1 expressing cells that co-express HLA-G (EVT) and decidual stromal cells, which do not express HLA-G.


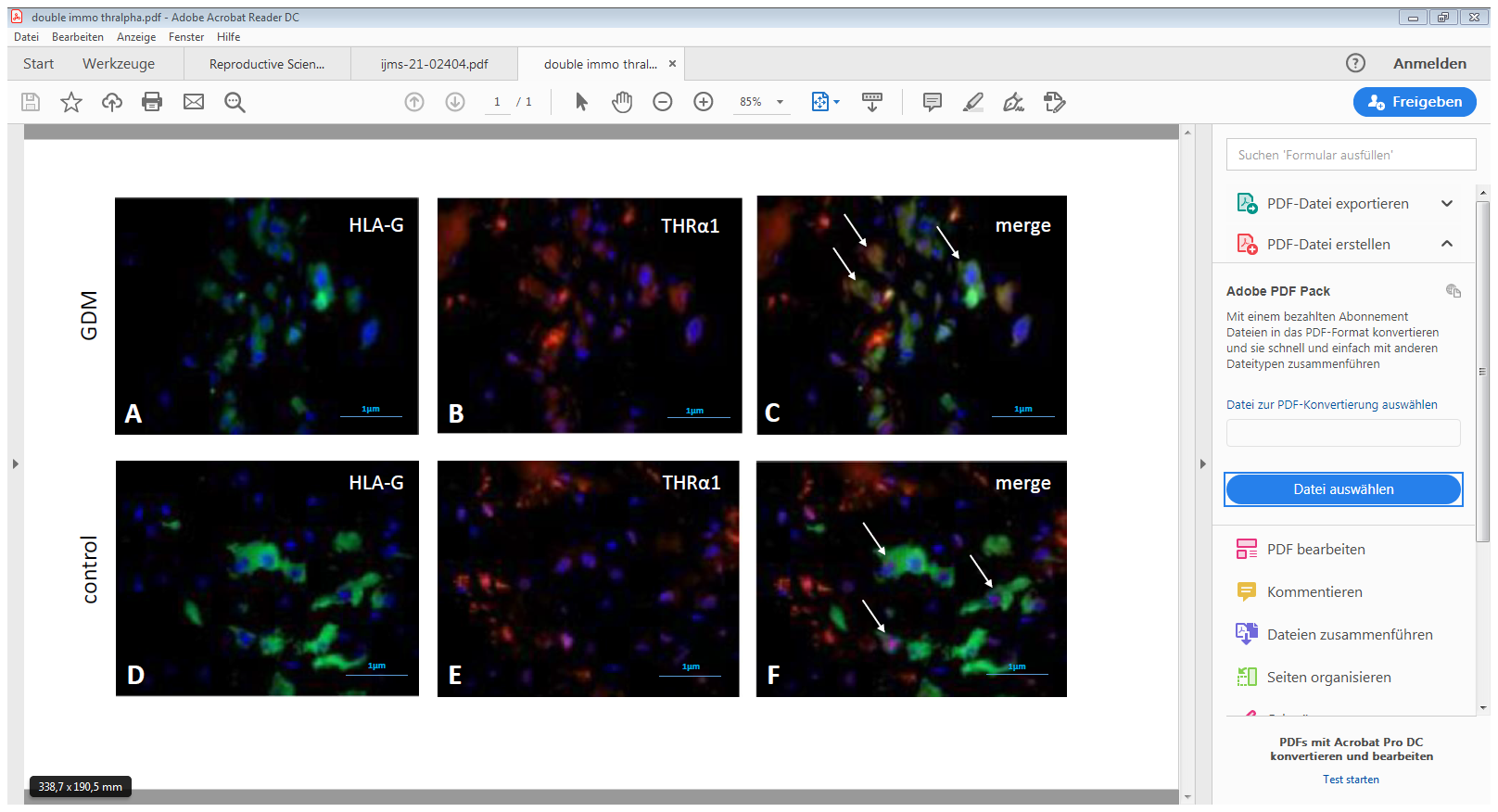


**Supplementary Figure S1:** Double immunofluorescence phenotyping of decidual cell. Nuclei are stained blue using DAPI. HLA-G, bound by Cy-2-labelled secondary antibody, stained green, marking the extravillous trophoblast. THRα1, bound by Cy-3-labelled secondary antibody, stained red. GDM:  GDM placental HLA-G staining of extravillous trophoblast cells is shown in (A). THRα1 staining in cells of the decidua is shown in (B). Triple filter excitation showing both, THRα1 and HLA-G is shown in (C). HLA-G staining of extravillous trophoblast cells in normal control trophoblast tissue is shown in (D). THRα1 staining of cells from the decidua from the same area is shown in (E). Triple filter excitation showing both, THRα1 and HLA-G is shown in (F). EVTs, which are positive for both HLA-G and THRα1 are marked with a white arrow.


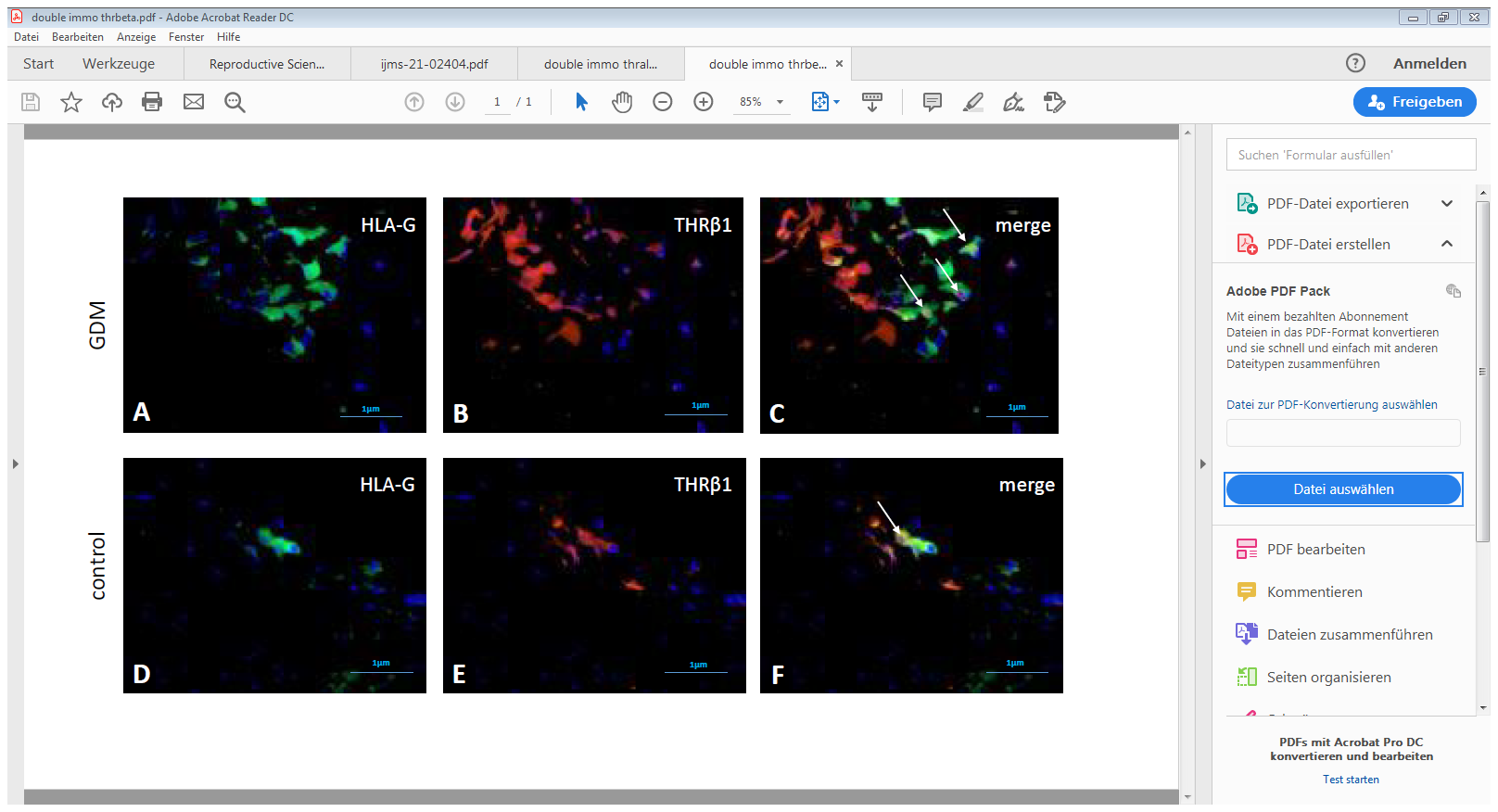


**Supplementary Figure S2:** Double immunofluorescence phenotyping of decidual cell. Nuclei are stained blue using DAPI. HLA-G expression, identified by Cy-2-labelled secondary antibody, stained green, marking the extravillous trophoblast. THRβ1, bound by Cy-3-labelled secondary antibody, stained red. GDM: HLA-G staining of extravillous trophoblast cells is shown in (A). THRβ1 staining in decidual cells from the same area is shown in (B). Triple filter excitation showing both, THRβ1 and HLA-G is shown in (C). White arrows mark cells expressing both antigens. Controls:  HLA-G staining of extravillous trophoblast cells is shown in (D). THRβ1 staining in decidual cells from the same area is shown in (E). Triple filter excitation showing both, THRβ1 and HLA-G is shown in (F).

**Methods:**

**Identification of THR expressing cells in the decidua with double immunofluorescence**

Double-immunofluorescence staining was performed in order to identify the cells expressing nuclear receptors THRα1 and THRß1 in the decidua, respectively. Double-immunofluorescence staining was carried out on placentas of GDM pregnancies and controls. For 20 min., the sections were deparaffinised in xylol and after washing them in ethanol they were incubated in ethanol/methanol again for 20 min. Next, the slides were rehydrated in an alcohol gradient and then placed in a pressure cooker with sodium citrate (pH=6.0). Washing of the slides in PBS followed and then the slides were blocked with ultra V blocking solution (Labvision) for 15 min. The slides were incubated with polyclonal anti-THRα/β rabbit IgG (**Table 2**) with an incubation time of 1 h. After that slides were incubated with monoclonal anti-HLA-G antibody. Sections were then incubated with the secondary antibodies. The slides were incubated with the Cy-3 labelled goat-anti-rabbit IgG antibody (Dianova), which was diluted 1:500, and the Cy-2-labelled goat-anti-mouse IgG antibody, diluted 1:100. Next, the slides were embedded in DAPI containing mounting buffer (Vector Laboratories). Afterwards, the slides were analysed with a fluorescent Axioskop photomicroscope (Zeiss, Oberkochen, Germany). Pictures were taken with a digital Axiocam camera system (Zeiss).
