# Supplementary figures and images for: Cell Type- and Sex-Specific Dysregulation of Thyroid Hormone Receptors in Placentas in Gestational Diabetes Mellitus
Source: Int J Mol Sci. 2020 Jun 5;21(11):4056. doi: 10.3390/ijms21114056 (PMC7313460; doi:10.3390/ijms21114056)

GDM

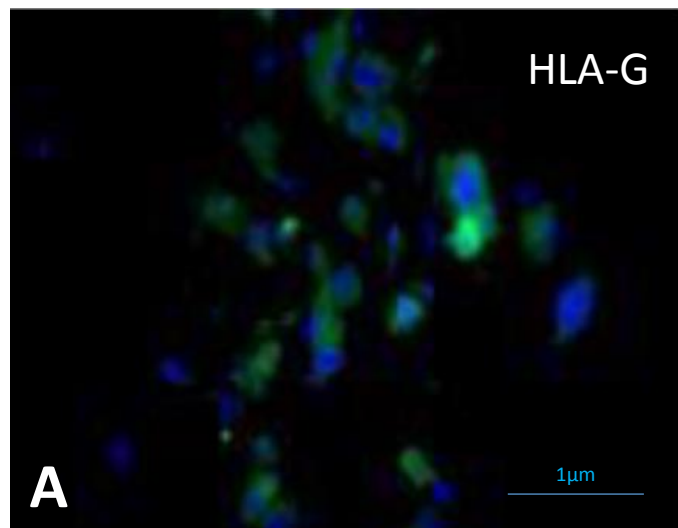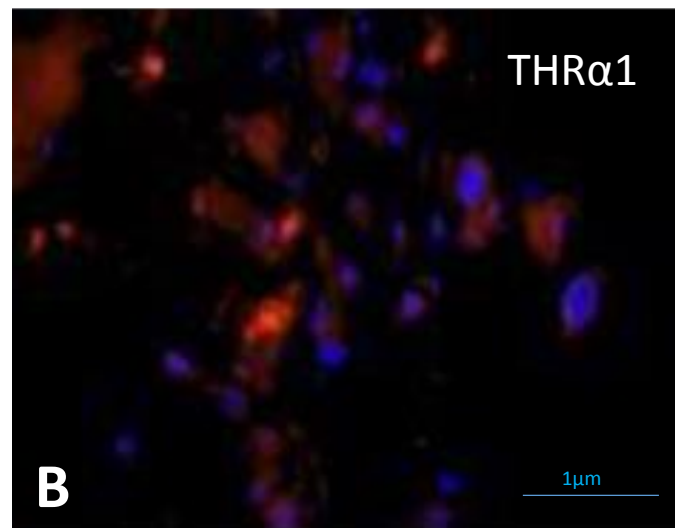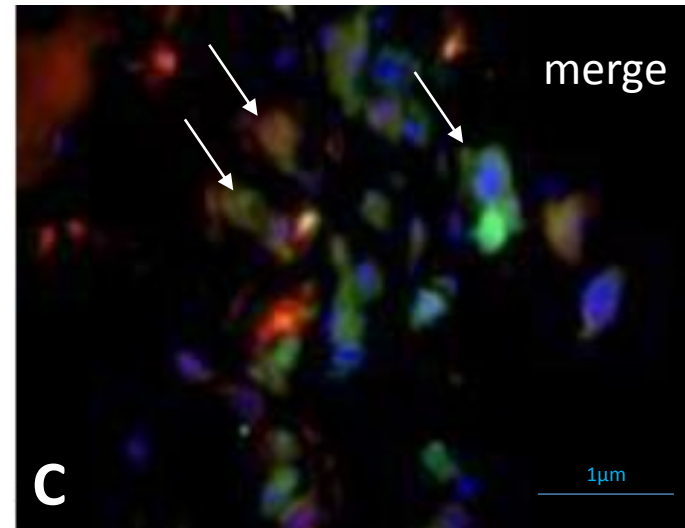

control

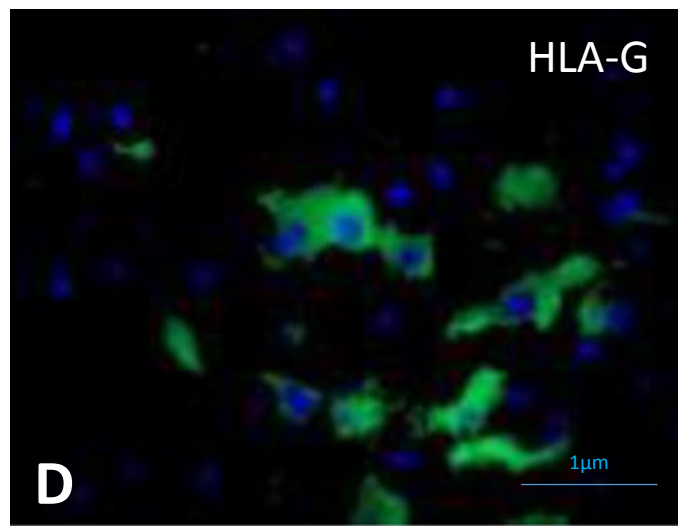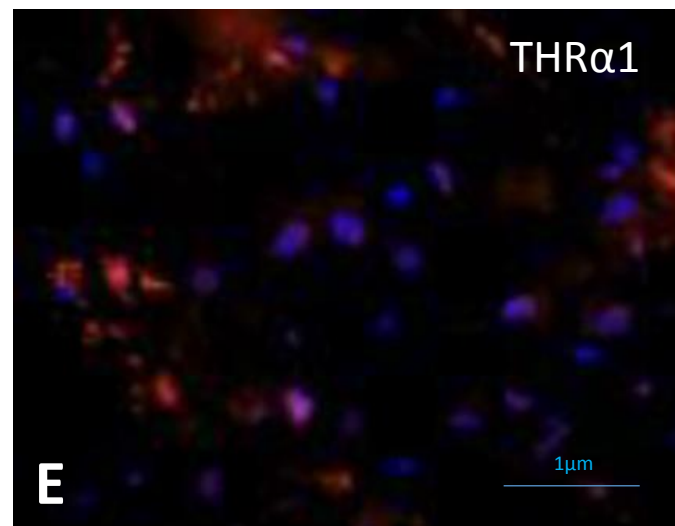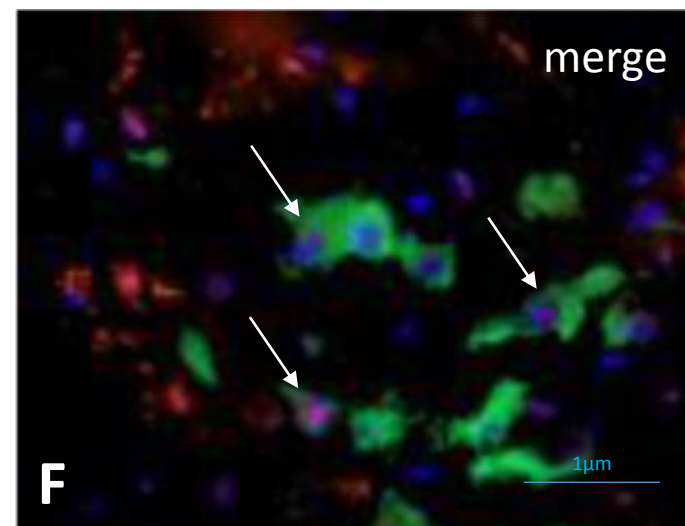

Supplement: Supplementary file 1 [file ijms-21-04056-s001.zip › supplementary material/Fig S1.pdf]

GDM

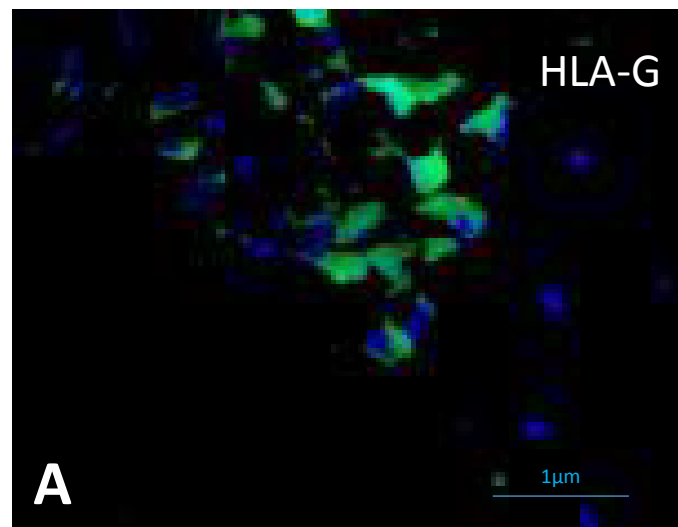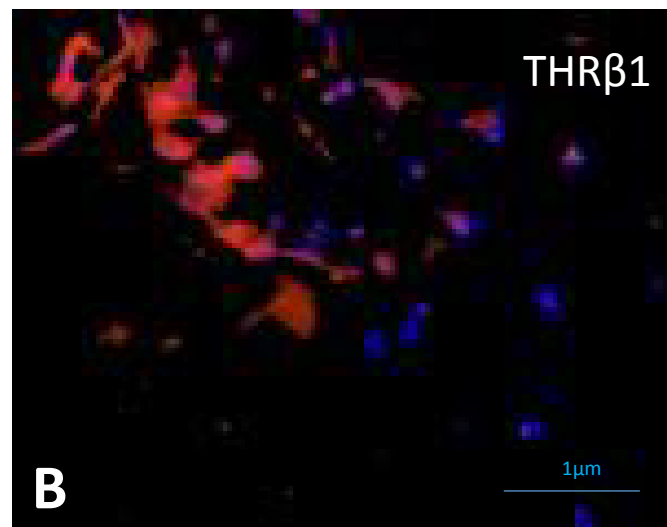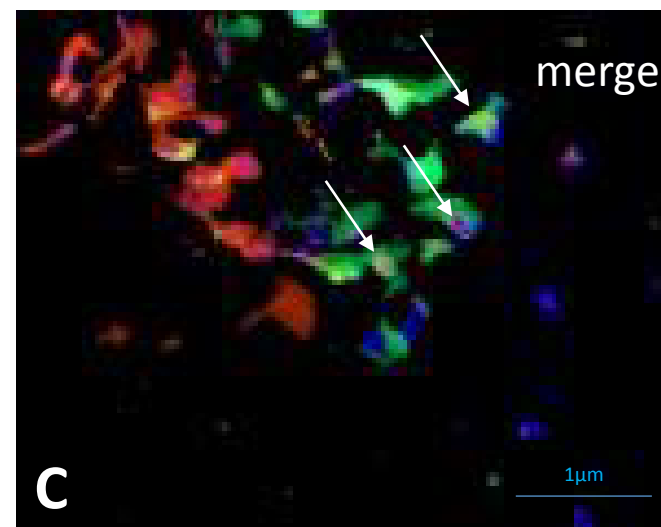

control

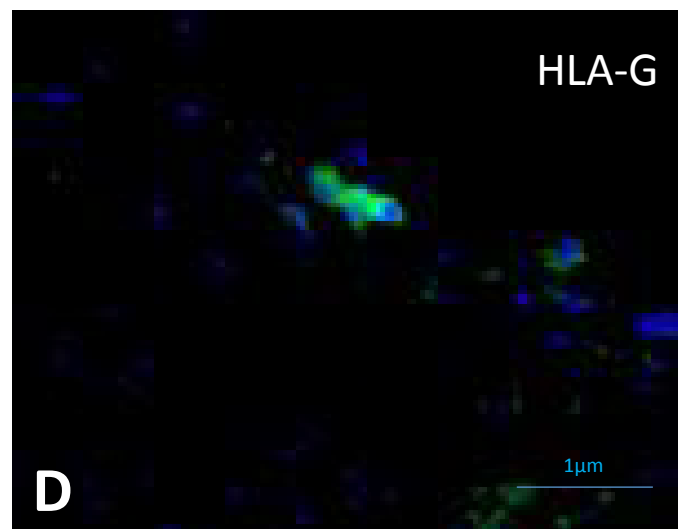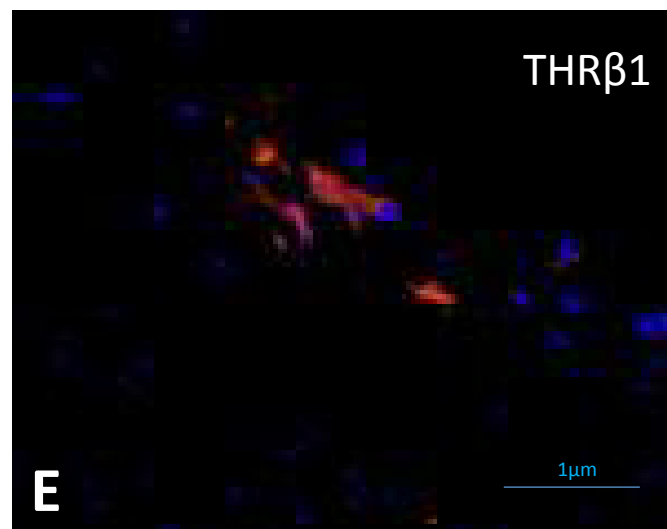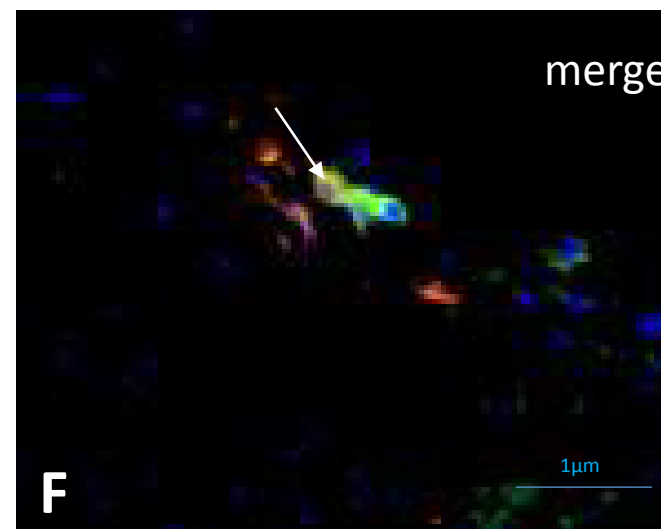

Supplement: Supplementary file 1 [file ijms-21-04056-s001.zip › supplementary material/Fig S2.pdf]
